# Cybersecurity considerations for radiology departments involved with artificial intelligence

**DOI:** 10.1007/s00330-023-09860-1

**Published:** 2023-07-07

**Authors:** Brendan S. Kelly, Conor Quinn, Niamh Belton, Aonghus Lawlor, Ronan P. Killeen, James Burrell

**Affiliations:** 1 Department of Radiology, St Vincent’s University Hospital, Dublin, Ireland; 2 Insight Centre for Data Analytics, UCD, Dublin, Ireland; 3 School of Medicine, University College Dublin, Dublin, Ireland; 4 Cybersecurity, Boston College, Boston, MA USA; 5 Information and Computer Science, University of Hawaii, Manoa, HI USA

**Keywords:** Radiology, Artificial intelligence, Cybersecurity

## Abstract

**Abstract:**

Radiology artificial intelligence (AI) projects involve the integration of integrating numerous medical devices, wireless technologies, data warehouses, and social networks. While cybersecurity threats are not new to healthcare, their prevalence has increased with the rise of AI research for applications in radiology, making them one of the major healthcare risks of 2021. Radiologists have extensive experience with the interpretation of medical imaging data but radiologists may not have the required level of awareness or training related to AI-specific cybersecurity concerns. Healthcare providers and device manufacturers can learn from other industry sector industries that have already taken steps to improve their cybersecurity systems. This review aims to introduce cybersecurity concepts as it relates to medical imaging and to provide background information on general and healthcare-specific cybersecurity challenges. We discuss approaches to enhancing the level and effectiveness of security through detection and prevention techniques, as well as ways that technology can improve security while mitigating risks. We first review general cybersecurity concepts and regulatory issues before examining these topics in the context of radiology AI, with a specific focus on data, training, data, training, implementation, and auditability. Finally, we suggest potential risk mitigation strategies. By reading this review, healthcare providers, researchers, and device developers can gain a better understanding of the potential risks associated with radiology AI projects, as well as strategies to improve cybersecurity and reduce potential associated risks.

**Clinical Relevance Statement:**

This review can aid radiologists’ and related professionals’ understanding of the potential cybersecurity risks associated with radiology AI projects, as well as strategies to improve security.

**Key Points:**

• *Embarking on a radiology artificial intelligence (AI) project is complex and not without risk especially as cybersecurity threats have certainly become more abundant in the healthcare industry.*

• *Fortunately healthcare providers and device manufacturers have the advantage of being able to take inspiration from other industry sectors who are leading the way in the field.*

• *Herein we provide an introduction to cybersecurity as it pertains to radiology, a background to both general and healthcare-specific cybersecurity challenges; we outline general approaches to improving security through both detection and preventative techniques, and instances where technology can increase security while mitigating risks.*

**Graphical Abstract:**

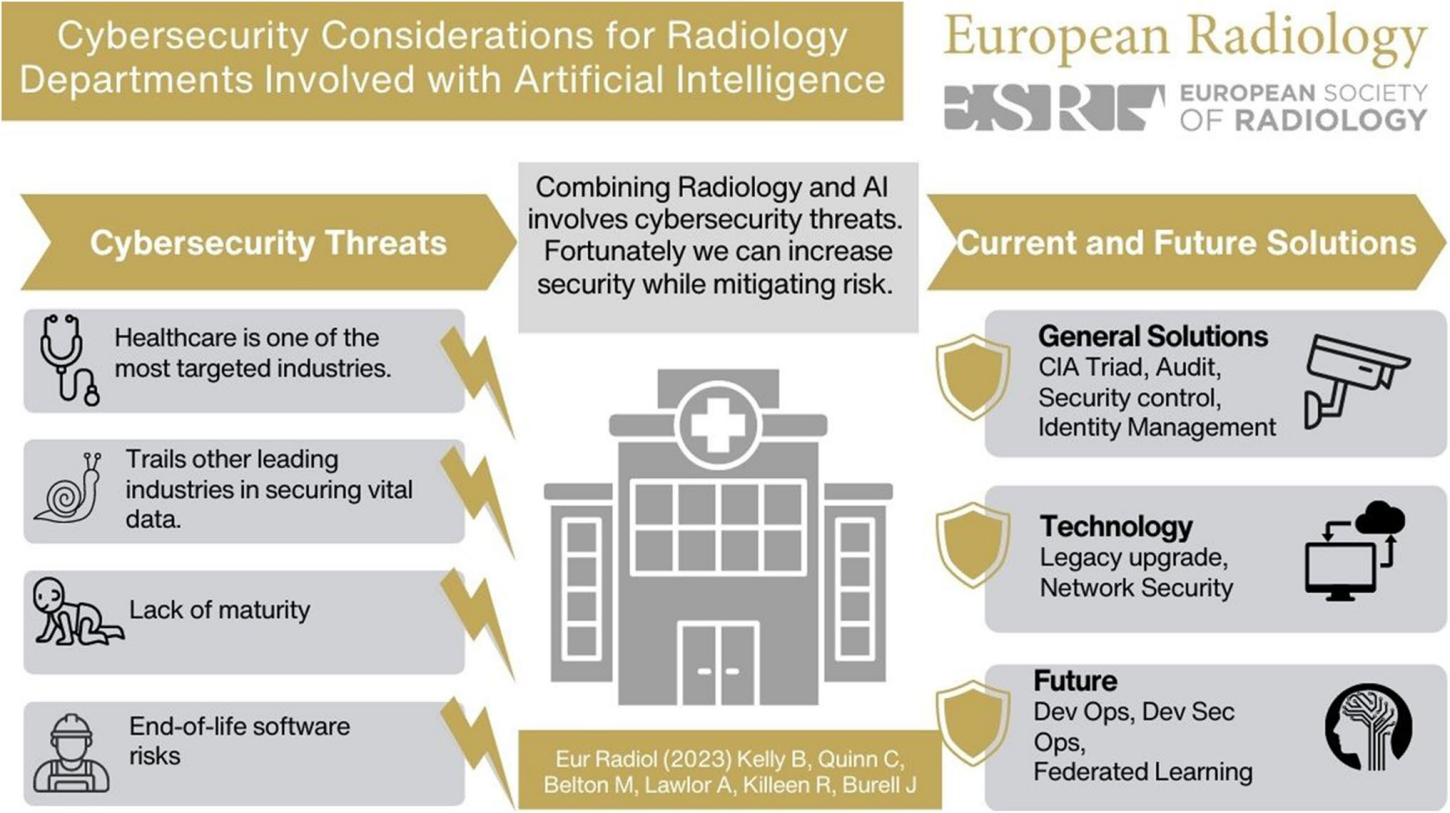

## Introduction

Embarking on a radiology artificial intelligence (AI) project includes the integration of a myriad of medical devices, wireless technologies, data warehouses, and even social networks. Cybersecurity threats are not entirely new to healthcare, but have certainly become more abundant with the advent of AI research in radiology and have been identified as one of the major healthcare risks of 2021 [1]. Healthcare providers and device manufacturers can benefit from studying cybersecurity practices in industry sector industries such as finance and defense, which have already implemented robust security measures to protect sensitive data and prevent cyber-attacks. By examining these approaches and adapting them to the unique challenges of the healthcare environment, providers and manufacturers can improve their cybersecurity posture and reduce the risk of data breaches and other security incidents in radiology AI projects. This review seeks to provide an introduction to cybersecurity as it pertains to imaging, a background to both general and healthcare specific cybersecurity challenges, general approaches to improving security through both detection and preventative techniques, and instances where technology can increase security while mitigating risks. We will first discuss general cybersecurity concepts and regulatory issues and then consider these topics as they pertain to radiology AI in terms of data, training, implementation, and auditability and finally suggest potential options to mitigate risks.

### General and regulatory considerations

#### General cybersecurity concepts

**Fig. 1:**
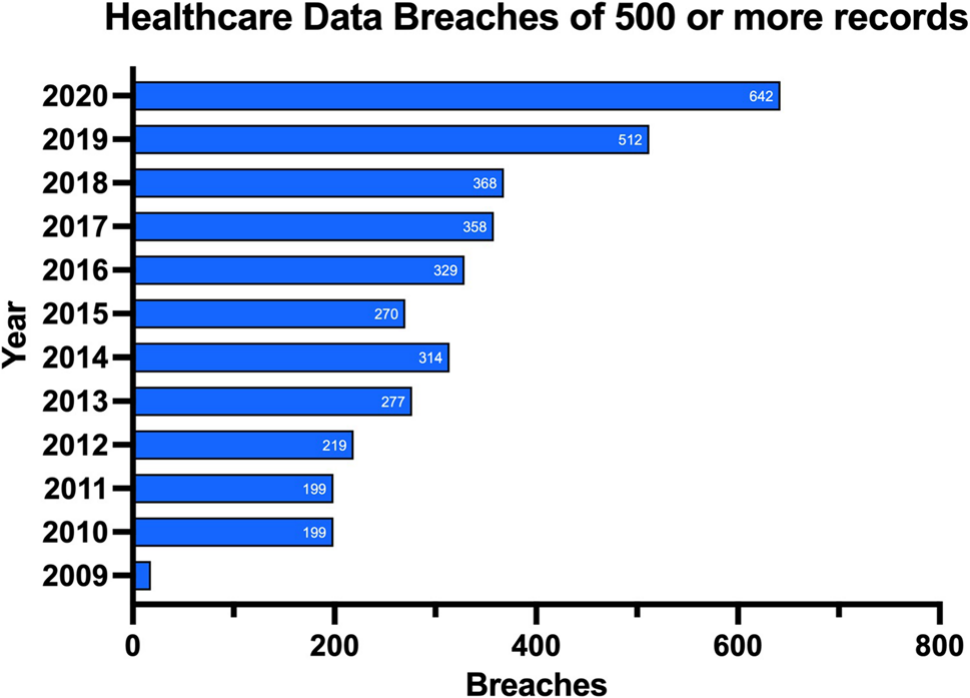
Number of data breaches reported to the Department of Health and Human Services’ Office for Civil Rights

The occurrences of data breaches and cyber-attacks such as ransomware have grown exponentially in recent years across all sectors [2]. Figure 1 shows how the number of data breaches reported to the Department of Health and Human Services’ Office for Civil Rights in the US has grown steadily from 2009 to 2020 [3]. As a result, mandatory, regulatory backed, cybersecurity programs are being implemented in organizations and are of particular importance in the healthcare industry due to the criticality of their role in society and the sensitivity of the data they retain. A well-known cybersecurity model that is used to define security programs within organizations is known as the “CIA Triad.” The CIA triad relates to the principles of confidentiality, integrity, and availability of data. Definitions of these terms are provided in (Table 1) [4].

**Table 1:** The CIA triad

Term	Definition
Confidentiality	Data is not disclosed (intentionally or unintentionally) to unauthorized individuals
Integrity	Data is not altered from its original state either accidentally or maliciously, by unauthorized individuals
Availability	Data is accessible and usable by authorized individuals

**Fig. 2:**
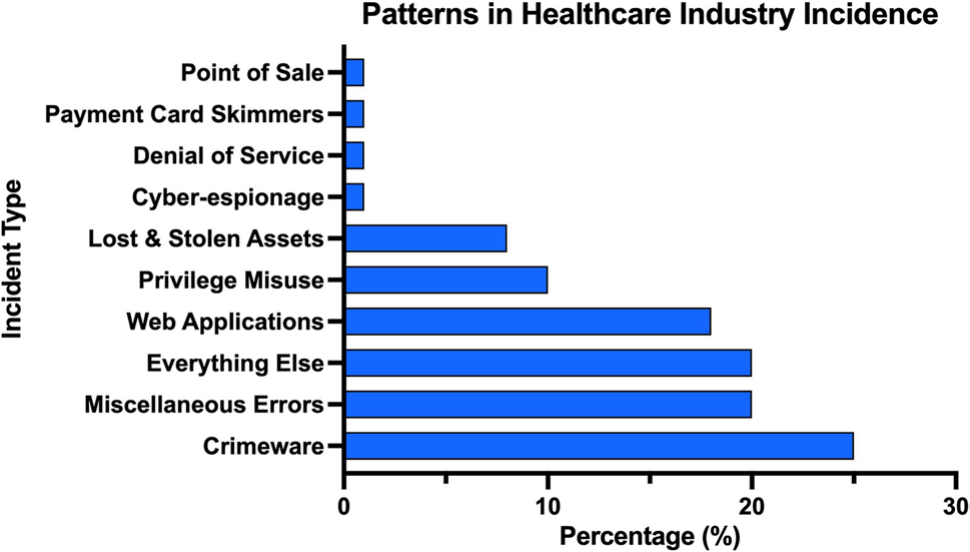
Patterns in healthcare industry incidents (*n* = 798)

**Fig. 3:**
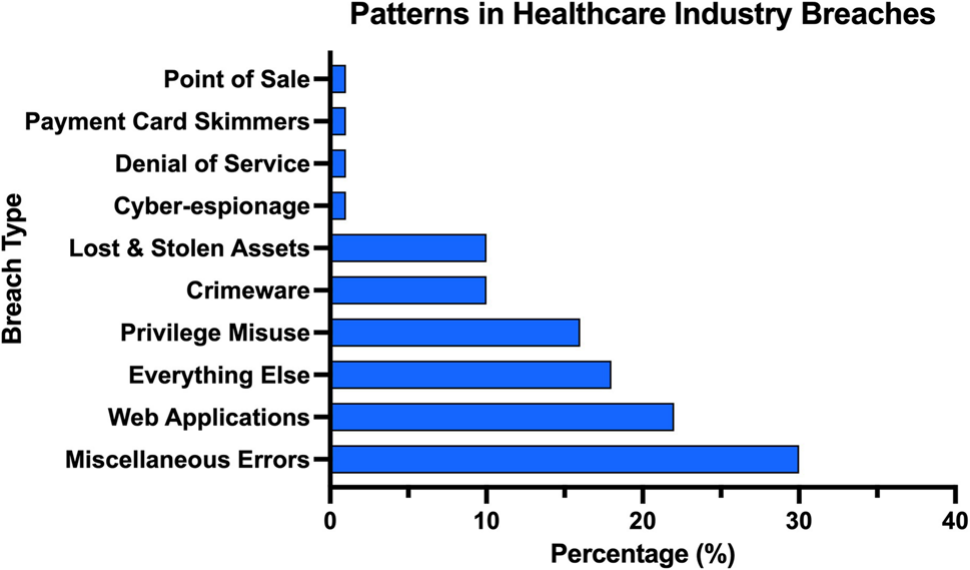
Patterns in healthcare industry breaches (*n* = 521)

**Fig. 4:**
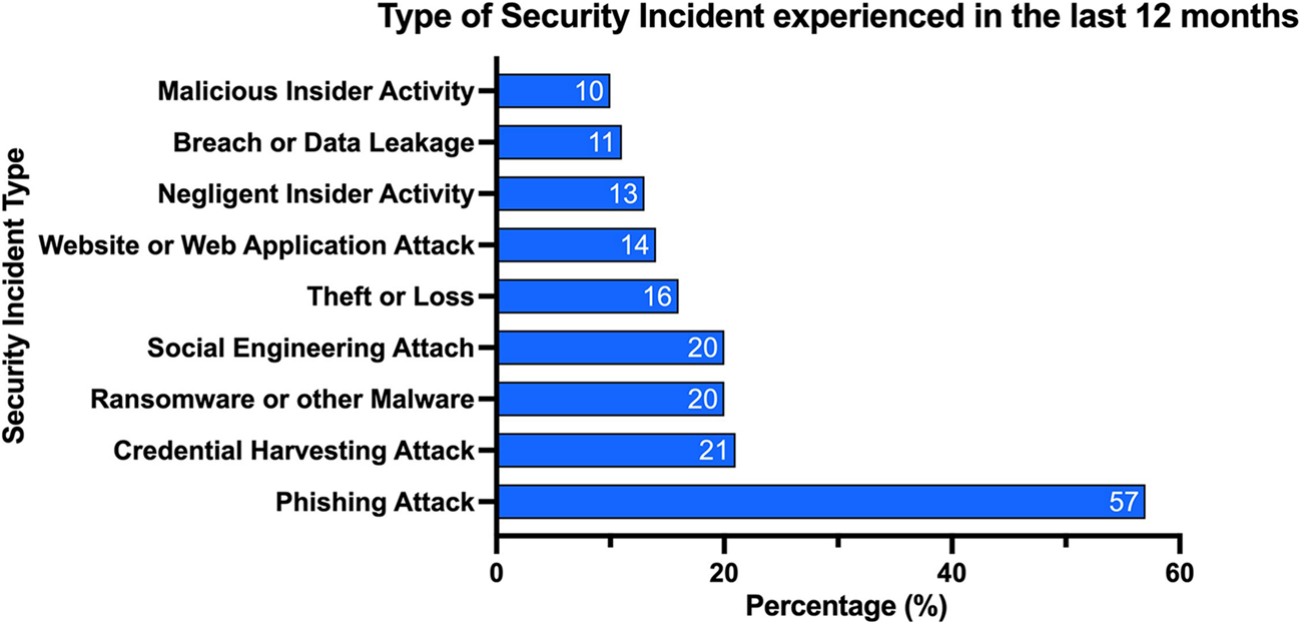
Type of significant security incident experienced in the past twelve months (*n* = 168)

**Fig. 5:**
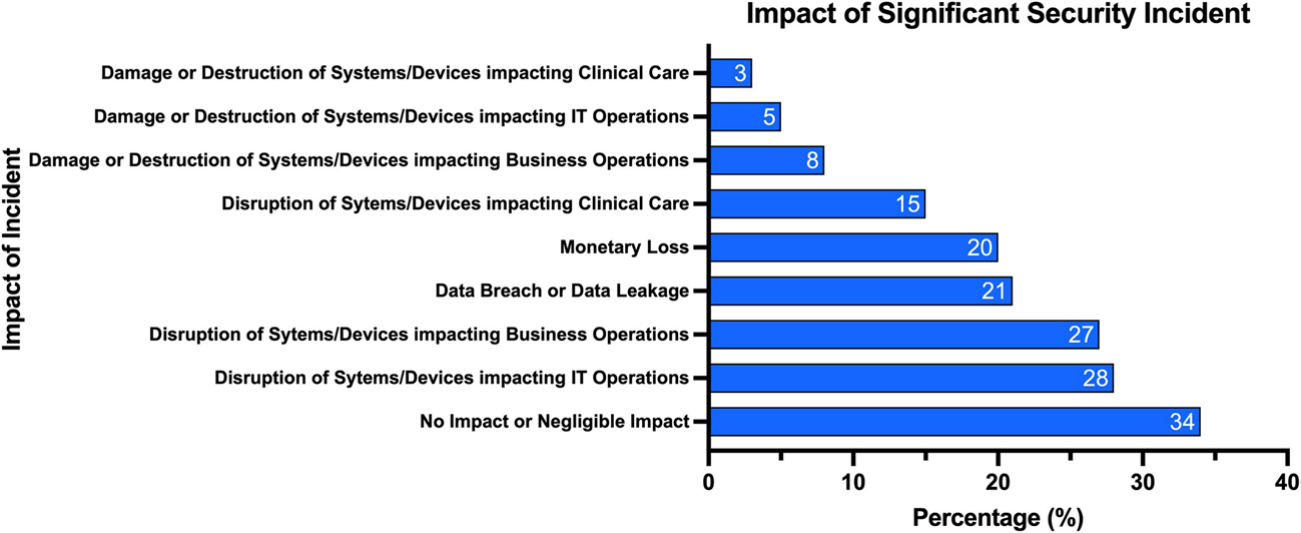
Significant security incidents—impact of incident (*n* = 168)

The healthcare industry is becoming a highly targeted industry for cyber-attacks. A survey of 223 organizations determined 81% were affected by cyber-attacks and over 110 million patients in the USA had their data compromised in 2015 [5]. Figure 2 shows the percentage of incident patterns by type and Fig. 3 shows the percentage of incidents by type [6]. Similarly, Fig. 4 shows the number of security incidents based on a survey of 168 US healthcare institutions as reported in the 2020 Healthcare Information and Management Systems Society (HIMSS) Cybersecurity Survey [7]. The healthcare industry is currently lagging behind other leading industry sectors in securing vital data [8]. The lack of maturity in healthcare cybersecurity programs can be a result of organizational risk prioritization, limited resourcing and financial backing, fragmented governance, and cultural behaviors. The consistent underinvestment in information technology (IT) can result in challenges to effectively secure legacy IT [8]. There is an elevated risk associated with the use of end-of-life software that has been clearly demonstrated in attacks on healthcare providers such as the Irish Health Service Executive “Conti” and the UK National Health Service “WannaCry” ransomware attacks [9, 10] (Fig. 5).

### Regulatory compliance and standards

Governments and regulators have responded to the rising number of cybersecurity threats by compelling organizations to implement robust cybersecurity programs to ensure all individuals’ personal information is protected. The U.S. Health Insurance Portability and Accountability Act (HIPAA) and the European Union General Data Protection Regulation (GDPR) [11, 12] are primary legislative actions that have resulted in improvement of cybersecurity programs.

#### HIPAA

HIPAA has a defined Security Rule that requires physicians to protect patients’ electronically stored, protected health information (known as “ePHI”). The rules require the physicians to use appropriate administrative, physical, and technical safeguards to ensure the confidentiality, integrity, and security of this information [11]. The Security Rule in HIPAA applies to any health care provider that transmits health information in electronic format.

#### GDPR

The GDPR does not apply specifically to cybersecurity or healthcare; it ensures that organizations store and process Personally Identifiable Information (PII) appropriately. GDPR is thus directly applicable to the healthcare industry. Concerning security, the GDPR “security principle” Article 5(1)(f) highlights the “integrity and confidentiality” of personal data. It states that personal data shall be:

*Processed in a manner that ensures appropriate security of the personal data, including protection against unauthorised or unlawful processing and against accidental loss, destruction or damage, using appropriate technical or organisational measures*.

Similar to HIPAA, GDPR does not specify particular security controls. However, security controls must be appropriate for the risk and protect the confidentiality and integrity of PII. The GDPR recognizes that it may be difficult or impractical for organizations to implement certain security measures. To address this issue, the GDPR includes the concept of “disproportionate effort,” which allows organizations to take into account the cost and technical feasibility of implementing certain security measures. Specifically, Article 32 of the GDPR states that organizations must implement appropriate technical and organizational measures to ensure the security of personal data, but these measures should be proportionate to the risks involved.

In other words, if the implementation of implementing certain security measures would require a disproportionate effort in terms of time, cost, or other resources, organizations may be allowed to implement alternative measures that still provide an appropriate level of security.

It is worth noting that the concept of disproportionate effort is not a loophole or an excuse for organizations to minimize the implementation of security measures. Rather, it is a recognition that there may be situations where implementing certain measures is not practical or feasible, and that organizations must assess and manage their risks in a way that is appropriate to their resources and capabilities.

A summary of the main regularity differences between the US and EU can be seen in Table 2 [13–15].

**Table 2:** Summary comparison table of HIPAA and GDPR

	GDPR	HIPAA
Protected data	Any data that relates to, or can lead to the identification of a living person	Any information about health status, care, or payment that is created or collected by a HIPAA Covered Entity (or a Business Associate of a Covered Entity), that can be linked to a specific individual
Scope	Sets compliance standards for all entities that fall within its scope, i.e., processing personal data	Sets standards for covered entities and their business associates (relating to medical data)
Security consideration	“..*Ensure appropriate security of the personal data, including protection against unauthorised or unlawful processing and against accidental loss, destruction or damage, using appropriate technical or organisational measures*”. ref2	*Require the physicians to use appropriate administrative, physical and technical safeguards to ensure the confidentiality, integrity and security of this information ref 3*
Data breach	The Supervisory Authority must be notified within 72 h. Affected persons must also be notified	Organizations must protect PHI and limit disclosure under the HIPAA Privacy Rule. Covered entities must also notify affected individuals of security breaches. If more than 500 people are affected, both affected individuals and the Department of Health must be informed within 60 days

### Data considerations

The last decade has seen a significant growth in the volume of medical data [16]. This creates a corresponding growth in the requirement for medical experts who can interpret the data. However, the supply of medical experts cannot currently meet the demand [17]. The use of AI has been suggested as one potential solution in order to address this issue [18]. The process of preparing data for medical imaging data for machine learning has been outlined which recognized ethics of data considerations related to the sharing of medical image data [19].

### Ethics of data

The ethical implications of using patients’ personal data for training and testing AI algorithms is complex and a matter for debate [20]. Larson and colleagues [21] argue that rather than considering if the healthcare institution or the patient “owns” the data, it is more effective to consider data as a resource that can benefit society. Kurpinski [22] has observed that almost every healthcare institution has had a third party request to purchase their data. While the ethics of buying data are still a subject for debate, it is clear that security and confidentiality updates are inextricably linked with good ethical standards.

### Access to data

The majority of digital heath data is stored in Electronic Heath Records (EHR). However, along with all the potential benefits are drawbacks related to privacy and security. One of the key potential security concerns relates to the number of different systems and services (radiology information system, labs, billing) that need to interact with the EHR that represent potential points of access. The ability for multiple users to interact with an EHR is a cornerstone of its value. Multiple users however pose risks of their own. Those with permissions to access the internet or download online content have the potential to be exploited.

### Querying data

The importance of different health care devices and information systems to freely exchange information is well established [23]. The framework for integration between different vendors and technologies is outlined in Health Level 7 (HL7). HL7 Version 3, which is based in XML, involves the transfer of text data without encryption. To facilitate transfer between different technologies, HL7 assumes that encryption will take place at a lower level, and provides no protocol level encryption.

### Data de-identification

De-identification removes or disassociates PII from patient data. This is an essential process to ensure patient privacy. However, there are several challenges involved in this process. For example, many types of software exist that will remove patient names and other data such as their date of birth from medical images. However, most medical images are complex files that contain metadata. This metadata can then be used to identify the patient. Moreover, the medical images themselves often contain identifiable information such as data that can be used to reconstruct a facial image of the patient. This emphasizes some of the additional complexities encountered by radiology specific use cases, the concept of disproportionate effort notwithstanding [24].

### Data storage

Digital Imaging and Communications in Medicine (DICOM) is the international 30-year-old standard protocol for managing and transmitting medical images, such as ultrasounds, MRIs, X-rays, and CT scans [25]. In 2019, researchers highlighted the possibility of “showing how an attacker can use deep learning to add or remove evidence of medical conditions from volumetric (3D) medical scans” (i.e., DICOM files) [26]. The researchers demonstrated that it was possible to use malware to alter the DICOM file format. There has been no such attack to date; however, this study highlights that security issues unique to the healthcare industry exist and have not been previously examined. This further emphasizes the need to examine healthcare technologies and industry standards to identify potential security vulnerabilities.

### Data transfer

Data is often required to be transferred to different healthcare institutions or providers. This transfer can be either physical or virtual via a network connection. Healthcare institutions often do not have the computational resources to perform large-scale data analysis and transfer the data to an external organization such as an industry partner to perform the data analysis. This process increases the cyber-attack surface. Whether data is stored locally or remotely, security and privacy is an important consideration for hardware and software applications, access control, and integrity of the systems governing these processes.

### Data labeling

Data labeling involves the assignment of one or more descriptors that provide context to data and represents one of the most interesting challenges both in Big Data and AI for digital health [27]. Data labels could become compromised during a cyber-attack. An AI algorithm may then be subsequently trained on this data which would result in inaccurate results or recommendations. This could have a harmful impact on patient care and may only become apparent during the evaluation or testing of the AI algorithm.

### Training

Machine learning systems are often unable to differentiate between valid and manipulated training input data. AI models are usually built using open-source platforms with Python, R, and C as the most popular coding languages for the development of AI algorithms [28]. Software that is developed in these coding languages are regularly targeted for attack. Moreover, machine learning projects often utilize open-source libraries such as Numpy and Scikit learn. The use of these libraries also pose security challenges. For example, it can be difficult to make quick changes to these libraries after a vulnerability is identified. Additionally, if several open-source libraries are used in the software, it can become challenging to identify all modules that are in use. This is especially the case when using high level APIs such as Keras and Pytorch. If all modules are not known, this can lead to unidentified vulnerabilities in the software. The algorithms themselves can pose cybersecurity challenges and convolutional neural networks, which is used in visual imagery analysis, have been shown to be susceptible to malicious attacks [29].

### Potential solutions

The maturity of cybersecurity programs within healthcare institutions may be at a level of concern; there are several recurring issues that will alleviate some risks.

#### Audit

Regular audit and clinical governance of systems are of paramount importance for the secure integration with digital health systems. This can be challenging for systems that use continuous learning as an approach to machine learning where a model is trained on a stream of incoming data over time, rather than on a fixed dataset. This allows the model to adapt to changes in the data distribution and continue to improve its performance over time [30]. Another major challenge is catastrophic forgetting which refers to the tendency of machine learning models to forget previously learned information when they are trained on new data [30]. Specifically, as a model learns new information, it may overwrite or “forget” previously learned information, leading to a degradation in performance on previous tasks. Catastrophic forgetting can be particularly problematic in scenarios where a model needs to perform multiple tasks over time, such as in healthcare or finance applications. For example, a model that is trained to diagnose a certain medical condition may need to be continuously updated with new data over time, but it also needs to retain its ability to diagnose previously seen conditions.

To address catastrophic forgetting in continuous learning, various techniques have been developed such as regularization methods, ensemble-based approaches, and generative replay. These methods attempt to balance the model’s ability to learn new tasks with its ability to retain previously learned information, allowing it to continuously improve its performance without forgetting what it has learned in the past.

#### Security controls

Strong security controls (such as encryption, multi factor authentication, and endpoint protection solutions) is an obvious first step in protecting data within healthcare institutions. In addition, organizations can align their information security programs to common cyber security industry frameworks to maintain a “minimum” level of security standards and controls. A well-known information security framework, ISO27001, is an international standard to manage information security, covering 14 differing control sets that cover a variety of information security domains, primarily focusing on the management of information security from a governance and policy level [31, 32]. In some circumstances, attestation of the security controls contained within the framework can be certified by external bodies to achieve certification for an organization’s security program. There is an interesting interplay between increasing security controls and compliance. Compliance with cyber security frameworks/regulatory requirements may appear to provide security; however, in practice, it may not provide an enhanced level of security and often struggle to maintain pace with technology advancement. Security controls are updated regularly and in some cases may provide greater protections than requirements specified by regulators.

#### Dealing with legacy software

As previously mentioned, due to the nature of technologies utilized within healthcare being unique to their environment, there can be a reliance on systems that are based on legacy software for operations. These systems often operate using legacy software (i.e., Microsoft Windows 7 operating system) that are at risk since they are not supported by the vendoro and do not have the latest security controls. If a vulnerability is discovered as part of a legacy software, it may not receive a fix and can become a permanent risk. Healthcare institutions should maintain a robust lifecycle and asset management program to determine the scope of their support infrastructure and identify the risks associated with legacy software. This also requires buy-in from healthcare technology vendors to ensure software is compatible with the latest operating system versions.

#### Network security

Healthcare systems use information systems to communicate administrative and clinical data between different departments and institutions. “Flat” networks allow devices on a network to communicate with other devices within the same network with very little routing/switching controls. It is known that using correct network segmentation when creating these communication paths significantly reduces the risk of a cyber-attack that could spread across the organization using these communication channels.

### Audit and logging

Medical devices are developed to serve a particular medical purpose accurately and safely. These devices should log all users’ actions on the devices. Therefore, when an incident occurs, the logs can be examined and a root cause for the incident can be identified. This data can then be used to improve the cybersecurity of the device and, hence, this will increase the overall security of the institution.

### Development operations and development security operations

As the occurrence of cyber-attacks increase and software development practices mature, the processes of development operations (DevOps) and development security operations (DevSecOps) for the development of software-based solutions have grown in popularity. These processes aim to shift the integration of cybersecurity earlier in the software development phase. This is known as “shifting left” and allows cybersecurity to be in-built to the software development process and allows for the identification of potential security concerns earlier in the process. Research has shown that implementing DevOps and DevSecOps processes result in more secure devices/systems [33]. Identifying security issues earlier in the development process also provides an opportunity for issues to be rectified earlier at a lower cost.

### Federated learning

Federated learning is an innovative technique that enables to development of more robust models [34]. Federated learning trains models locally in hospitals and institutions, instead of collecting the data from different institutions and storing the data centrally. Hence, sensitive data can remain with its data controller, avoiding the associated legal and security complications of transmitting data between institutions.

A common federated learning approach is federated averaging, where clients are AI models working locally on each site’s data. They independently perform local gradient updates and transmit their weights back to a central server. The weights across all sites are averaged and broadcasted back to all sites for the next round of training.

Tuning of pre-trained weights on another site’s data is another common collaborative technique. However, this technique is usually applied when the weights trained from one site are easily accessible, such as from a public datasets [35]. In Cyclical Institutional Incremental Learning and Institutional Incremental Learning techniques, one model is passed from one site to another until the data from all sites is used to incrementally update the weights of the model.

Learning can be performed multiple times to ensure that earlier features are constantly incremented. Federated averaging requires synchronization and communication to a server from many clients, which can become a significant overhead during the training procedure. However, conceptually, federated averaging is more robust for a network of sites that may contain a limited number of adversaries that may try, perhaps unknowingly, to slow down the network or inject bad or skewed data [36].

Another challenge for federated learning is the points of access for a cyber-attack increase with the number of nodes. If a single node is compromised, the integrity of the model will be impacted. It is a challenging task to ensure all systems are consistently updated with the latest security controls. The federated learning model can introduce particular security weaknesses that grow exponentially as the potential attack surface increases with each additional node.

## Conclusion

In this review, we have provided a background to cybersecurity as it pertains to radiology artificial intelligence. We have introduced the core concepts and how they apply to data training and implementation as well as provided an introduction to potential methods to mitigate risk. While the potential for AI to revolutionize the practice of radiology is clear, it is important to realize the potential impact of increased connectively and adoption of technology on the confidentiality, integrity, and availability of healthcare data.

## Abbreviations

AI: Artificial intelligence
DevOps: Development operations
DevSecOps: Development security operations
DICOM: Digital Imaging and Communications in Medicine
EHR: Electronic heath records
ePHI: Electronically stored Protected Health Information
HL7: Health Level 7
HIMSS: Healthcare Information and Management Systems Society
HSE: Irish Health Service Executive
PII: Personally Identifiable Information
GDPR: The European Union General Data Protection Regulation
HIPAA: The U.S. Health Insurance Portability and Accountability Act
NHS: UK National Health Service

## Declarations

### Guarantor

The scientific guarantor of this publication is Ronan P Killeen.

### Statistics and biometry

No complex statistical methods were necessary for this paper.

### Methodology

• multicenter study
